# Molecular diagnoses and candidate gene identification in the congenital heart disease cohorts of the 100,000 genomes project

**DOI:** 10.1038/s41431-024-01744-2

**Published:** 2024-11-26

**Authors:** Verity Hartill, Mitra Kabir, Sunayna Best, Wasay Mohiuddin Shaikh Qureshi, Stephanie L. Baross, Jenny Lord, Jing Yu, Erina Sasaki, Hazel Needham, Deborah Shears, Matthew Roche, Elizabeth Wall, Nicola Cooper, Gavin Ryan, Jacqueline Eason, Robert Johnson, Bernard Keavney, Kathryn E. Hentges, Colin A. Johnson

**Affiliations:** 1 Leeds Institute of Medical Research, University of Leeds, St James University Hospital, Beckett Street, Leeds, UK; 2 Yorkshire Regional Genetics Service, Leeds Teaching Hospitals NHS Trust, Chapel Allerton Hospital, Leeds, UK; 3 Division of Evolution, Infection and Genomics, School of Biological Sciences, Faculty of Biology, Medicine and Health, University of Manchester, Michael Smith Building, Oxford Road, Manchester, UK; 4 Division of Cardiovascular Sciences, Faculty of Biology, Medicine and Health, The University of Manchester, Manchester, UK; 5 Department of Human Development and Health, Faculty of Medicine, University of Southampton, Southampton, UK; 6 Novo Nordisk Research Centre Oxford, The Innovation Building, Roosevelt Dr, Headington, Oxford, OX3 7FZ UK; 7 Oxford Centre for Genomic Medicine, ACE Building, Nuffield Orthopaedic Centre, Oxford University Hospital NHS Foundation Trust, Oxford, UK; 8 Dingley Specialist Childrens Centre, University of Reading Campus, Royal Berkshire NHS Foundation Trust, Berkshire, UK; 9 Windsor House Group Practice, Leeds, UK; 10 West Midlands Regional Genetics Service, Birmingham Women’s and Children’s Hospital, Birmingham, UK; 11 West Midlands Regional Genetics Laboratory, Birmingham Women’s Hospital, Mindelsohn Way, Birmingham, UK; 12 Nottingham Regional Genetics Service, City Hospital, Nottingham, UK; 13 Alder Hey Children’s NHS Foundation Trust, Eaton Road, Liverpool, UK; 14 Manchester Heart Institute, Manchester University NHS Foundation Trust, Manchester Academic Health Science Centre, Manchester, UK

**Keywords:** Genetics research, Medical genomics

## Abstract

Congenital heart disease (CHD) describes a structural cardiac defect present from birth. A cohort of participants recruited to the 100,000 Genomes Project (100 kGP) with syndromic CHD (286 probands) and familial CHD (262 probands) were identified. “Tiering” following genome sequencing data analysis prioritised variants in gene panels linked to participant phenotype. To improve diagnostic rates in the CHD cohorts, we implemented an agnostic de novo Gene Discovery Pipeline (GDP). We assessed de novo variants (DNV) for unsolved CHD participants following filtering to select variants of interest in OMIM-morbid genes, as well as novel candidate genes. The 100kGP CHD cohorts had low rates of pathogenic diagnoses reported (combined CHD “solved” 5.11% (*n* = 28/548)). Our GDP provided diagnostic uplift of nearly one third (1.28% uplift; 5.11% vs. 6.39%), with a new or potential diagnosis for 9 additional participants with CHD. When a filtered DNV occurred within a non-morbid gene, our GDP prioritised biologically-plausible candidate CHD genes (*n* = 79). Candidate variants occurred in both genes linked to cardiac development (e.g. *AKAP13* and *BCAR1*) and those currently without a known role (e.g. *TFAP2C* and *SETDB1*). Sanger sequencing of a cohort of patients with CHD did not identify a second de novo variant in the candidate dataset. However, literature review identified rare variants in *HMCN1*, previously reported as causative for pulmonary atresia, confirming the approach utility. As well as diagnostic uplift for unsolved participants of the 100 kGP, our GDP created a dataset of candidate CHD genes, which forms an important resource for further evaluation.

## Introduction

Congenital heart disease (CHD) is a structural cardiac defect present from birth, occurring in approximately 1 in 100 live births [1]. It comprises a wide range of conditions including septal, valvular and outflow tract lesions. CHD can be non-syndromic (as an isolated feature) or syndromic (associated with other congenital anomalies) [2].

CHD often occurs as a sporadic event, where the cause is largely unknown, but is suggested to be either heterogenic or the result of a genetic-environmental interaction [3]. However, in families with a sporadic case, there is an increased familial recurrence rate of approximately threefold across all CHD conditions, indicating a genetic basis. The genetic aetiology of sporadic CHD is known to be highly complex; in addition to rare variants of large effect (eg [4]), copy number variants (eg [5]) and common SNPs (eg [6]) all affecting the risk of CHD. Even with the large number of genes now associated with monogenic forms of CHD, these causes account for less than one third of all cases [3].

The 100,000 Genomes Project (100kGP) was a clinical research project launched in 2012 and overseen by Genomics England (GEL), a company owned by the UK Government Department of Health and Social Care [7, 8]. The project aimed to sequence genomes of 85,000 National Health Service (NHS) participants to provide insights into the role of genomics in healthcare.

Within the 100kGP, there were two categories for recruitment for CHD participants; syndromic and familial CHD. For “syndromic CHD”, 100kGP eligible participants had CHD with no acquired antenatal cause or recognised syndrome AND greater than or equal to one feature of: extra cardiac malformation OR neurodevelopmental delay. For “familial CHD”, participants had CHD with no acquired antenatal cause or recognised syndrome AND: either ≥1 affected first-degree relative OR parental consanguinity [7].

Variants identified following genome sequencing (GS) of rare disease participants in the 100kGP were “Tiered” by the GEL pipelines into three Tiers according to predicted pathogenicity. An automated pipeline filtered down to rare, segregating, and functionally important variants, followed by a virtual panel-based approach to select known morbid genes [7]. Gene panels were applied according to the recruitment categories and Human Phenotype Ontology (HPO) terms submitted by the referring clinician [7]. NHS clinical laboratories were supported by GEL to assess variants classified as Tiers 1 and 2, but not for Tier 3 or un-tiered variants [7].

Participants recruited to the 100kGP CHD cohorts had a relatively low reported “solved” rate from the project. Our initial cohort study indicated that the syndromic CHD “solved” rate was 6.29% (*n* = 18/286), and familial CHD was 3.89% (*n* = 10/262). This is low when compared to other cohorts, for example intellectual disability (“solved” rate 21.4% (*n* = 1425/6664)) or unexplained skeletal dysplasia (“solved” rate 17.6% (*n* = 45/255)).

De novo variants (DNVs) are an important cause of CHD in both syndromic and non-syndromic cases, as had been demonstrated in numerous previous studies [9, 10]. We created a de novo Gene Discovery Pipeline (GDP) to identify DNVs in GS data from participants recruited to the sCHD and fCHD cohorts of 100kGP to provide diagnostic uplift. Participants from both data-sets were included, even though DNVs may not be a common cause of disease in familial or inherited CHD. However, we hypothesised that DNVs could still have an important role in these cases, for example if familial heterogeneity or gonadal mosaicism was present.

## Materials and methods

### Identification of 100,000 genomes project participants

Participant data was accessed through the secure GEL “Research Environment” (RE) [8]. Participant recruitment category, clinical data, HPO terms, panels applied and project outcomes were accessed through the “LabKey” data management software (main programme v14 accessed 27/01/2022). Participant’s “solved” status was assessed according to the Genomic Medicine Centre (GMC) “Exit Questionnaire.” Further clinical data was extracted from GEL’s “Participant Explorer,” also accessed through the RE.

### Assessment of “solved” and “unsolved” participants

**Fig. 1:**
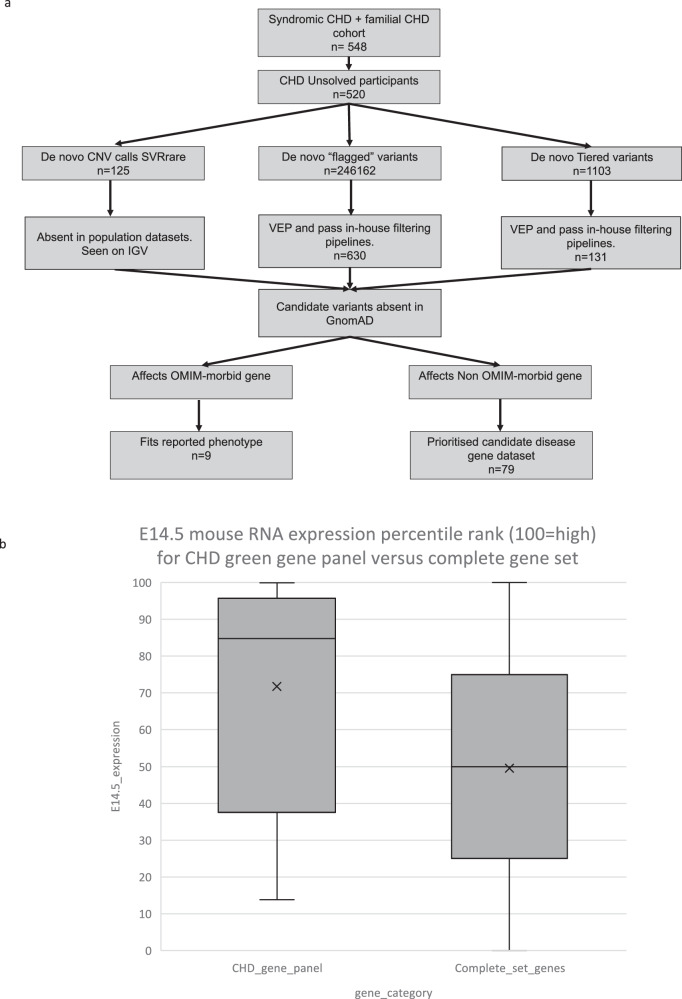
Gene Discovery Pipeline workflow used in this study and comparison of E14.5 mouse RNA expression rank score between genes on the “Familial non-syndromic congenital heart disease” gene panel and the full set of reported genes from the mouse genome. **a** Gene discovery pipeline schematic showing the process of de novo variant filtering pipelines. If filtered variants occurred within OMIM morbid genes that were compatible with the participant phenotype, then they were reported to the recruiting clinical team. If filtered variants occurred in OMIM non-morbid genes, they formed part of the candidate gene dataset. Numbers given indicate the number of participants or variants remaining after each stage of filtering. **b** Percentile rank expression from mouse E14.5 developing heart. A subset of genes known to be causative of CHD, which were “green genes” (*i.e*. validated genes included on the panel) from the “Familial non-syndromic congenital heart disease” gene panel, were shown to have a significantly higher mean expression percentile rank score (mean 71.79, SD 30.9), than the full set of reported genes from the mouse genome (mean 49.53, SD 29.6) (*t*(17702, total number of genes) = 3.602, unpaired *t*-test *p* < 0.01). Box plots are shown; horizontal lines = first quartile, median and third quartile values, crosses = mean values and error bars = maximum and minimum values.

Participants with coded “solved” status (i.e. a pathogenic variant consistent with the phenotype had been identified) within the identified CHD cohorts were highlighted and removed from further analysis. Participants coded as “unsolved”, “partially solved”, “report not available” and “unknown” from the CHD cohorts are hereafter termed “CHD unsolved participants”. GS data from “CHD unsolved participants”, where trio GS data were available, were assessed further by our GDP (Fig. 1a).

### Gene discovery pipeline

#### Variant identification methods

##### De novo “flagged” variants

All trios analysed by the GEL rare disease interpretation pipeline, based on Data Release v9 (02/04/2020), had genomes aligned by the Illumina iSSAC aligner and a multi-sample .VCF file was created per family using the Platypus variant caller. GEL performed a DNV annotation pipeline that “flagged” likely DNVs for each trio based on an array of filters that interrogated the multi-sample .VCF outputs. For each family, the multi-sample .VCF was fed into the Platypus “bayesiandenovofilter.py” Python script, run with default parameters. The custom DNV annotation script (process_denovo.R) was run per trio using this multi-sample .VCF as input. This script “flags” putative DNVs based on a pre-defined set of filters. A LabKey table (“denovo_flagged_variants”) included all variants that passed the “base_filter”; i.e. for autosomes, those that passed “zygosity filter” (genotype heterozygous in proband but homozygous reference in the parents), “mindepth filter” (minimum read depth 20x in offspring and both parents) and “maxdepth filter” (maximum depth of 98x in the offspring) for all trios within the DNV dataset. Variants in the “denovo_flagged_variants” dataset were identified for those occurring in samples from the “CHD unsolved participants” cohort and taken forward for further analysis.

##### Tiered variants

The “CHD unsolved participants” cohort were analysed to identify all Tier 1, 2 and 3 DNVs, and these variants were taken forward for further analysis.

##### SVRare

Analysis was performed using the SVRare tool as previously described [11, 12]. SVRare uses a database of 554,060 structural variants (SVs) called by Manta [13] and Canvas [14] copy number callers, collated from 71,408 participants in the rare disease arm of 100kGP. In this study, structural variants (SVs) called by Manta and Canvas from “CHD unsolved participants” were retained if they were de novo in the proband and passed the internal SVRare filters. Variants were annotated to show their presence in genes from the following GEL 100kGP panels: “laterality disorders and isomerism” OR “familial non-syndromic congenital heart disease”; and were annotated using pLI score, HI score and occurrence in other samples within the 100kGP. Identified variants were manually assessed to identify those rare in both other samples within the 100kGP (excluded if the call occurred in >10 100kGP participants) and control population datasets (Database of Genomic Variants [15]). Structural variants in .BAM files were confirmed using the Integrative Genomics Browser (IGV) [16]. SVs were considered potentially causative if present in >30% of reads.

#### Variant filtering

DNVs identified using GDP methods a) and b) as described above, were annotated with the Variant Effect Predictor (VEP) using Python scripts separately for participants who had GS data aligned to either GRCh37 or GRCh38. VEP Plug-ins were used to add annotations for SpliceRegion, CADD score and SpliceAI delta score. An in-house Python script (“filter_vep_output_variants.py”) was used to identify variants that were: termed “pathogenic” OR “likely pathogenic” by ClinVar; OR “high impact” (variants termed high impact by VEP annotation; stop_gained, stop_lost, start_lost, splice_acceptor_variant, splice_donor_variant, frameshift_variant, transcript_ablation, transcript_amplification); OR missense variants with a CADD score >15; OR variants with SpliceAI delta score >0.5. All candidate DNVs were confirmed to be absent from the gnomAD database [17].

#### Assessment of filtered variants

If DNVs occurred within OMIM morbid genes (genes with a confirmed OMIM morbid association), the participant phenotype was assessed alongside the genotype. If the HPO terms listed were compatible with the disease gene, the variant was reviewed and pathogenicity was assessed according to current American College of Medical Genetics (ACMG) guidelines. Once an informative variant was identified, the recruiting clinician was contacted using the GEL Airlock system, after review by the Airlock Committee, to inform them of the finding. To protect participant identity, the HPO terms given here are raised up one level in the HPO hierarchy. Additional clinical information and participant consent for publication was requested where required.

If output DNVs occurred within a novel non-morbid gene, this variant remained as part of the candidate CHD disease gene dataset. Genes in the candidate dataset were manually annotated to allocate key characteristics and enable prioritisation. Annotation was undertaken using LOEUF score [17], GTEX expression data (Heart; left ventricle, TPM) from deceased human individuals [18], accessed 7 Mar 2023, and data from RNA expression from developing heart in mice at embryonic day (E) 14.5 (FPKM, expressed as ranked centiles, see [10]). The mouse expression data was derived from RNA sequencing of tissue from E14.5 mouse embryos (for full methodology see [19]). The datasets used for this study were from left and right atria, left ventricle (with interventricular septum, aortic and mitral valves), and right ventricle (with pulmonary and tricuspid valves). The average of rpm (reads assigned per million mapped reads) of each gene from each chamber was used as the overall measure of heart expression.

To ensure the utility of the methodology, a subset of “green” genes (i.e. those genes included on the panel) from the “Familial non-syndromic congenital heart disease” panel used by Genomics England, were also manually annotated using the mouse E14.5 expression data [10]. These genes had a significantly higher mean expression rank score (mean 71.79, SD 30.9) than the full set of reported genes from the mouse genome (mean 49.53, SD 29.6) (*t*(17702, total number of genes)=3.602, unpaired *t*-test *p* < 0.01) (Fig. 1b). Candidate gene function was manually assessed using the OMIM database ([20] accessed March 2023), and a PubMed literature search ([21] accessed March 2023). Statistics and box plots were calculated using IBM SPSS Statistics for Macintosh, version 28.0.1.1 [14].

### Assessment of candidate variants in patients affected with CHD and their unaffected parents

An additional cohort of 1408 patients affected with CHD, for whom singleton whole exome sequencing (WES) data was available, were assessed for variants in the full list of candidate genes. The cohort was previously described in [4, 22] and included 818 patients affected with tetralogy of Fallot (TOF), and 578 patients with mixed CHD. Sequencing was performed on Illumina NovaSeq machines generating 150 bp paired-end reads using the Agilent SureSelect Human All Exon exon capture kit (v4 for TOF samples, v6 for mixed CHD). Sequencing data was aligned to GRCh38 using BWA-MEM (v0.7.15), variants called with WeCall (v2.0.1), and annotated using Ensembl VEP (v93). Variants were retained that were: absent in gnomAD; AND were “high impact” (variants termed high impact by VEP annotation) OR missense variants with a CADD score ≥15. In total there were 186 variants identified: 24 were high impact, and 162 were missense variants with CADD score ≥15. Twelve variants for which unaffected parental samples were available were selected for Sanger confirmation and segregation in parents (Supplementary Fig. 1). The variant region of interest was amplified using PCR. The band was excised from agarose gel, DNA was extracted (NEB gel extraction kit) and sent for sanger sequencing (Eurofins Genomics). The resulting sequences were subjected to BLAT analysis for alignment to human reference genome (GRCh38/hg38), and the obtained chromatograms were analysed using SnapGene software.

## Results

### The majority of participants recruited to the CHD cohorts were not solved by GEL pipelines

**Fig. 2:**
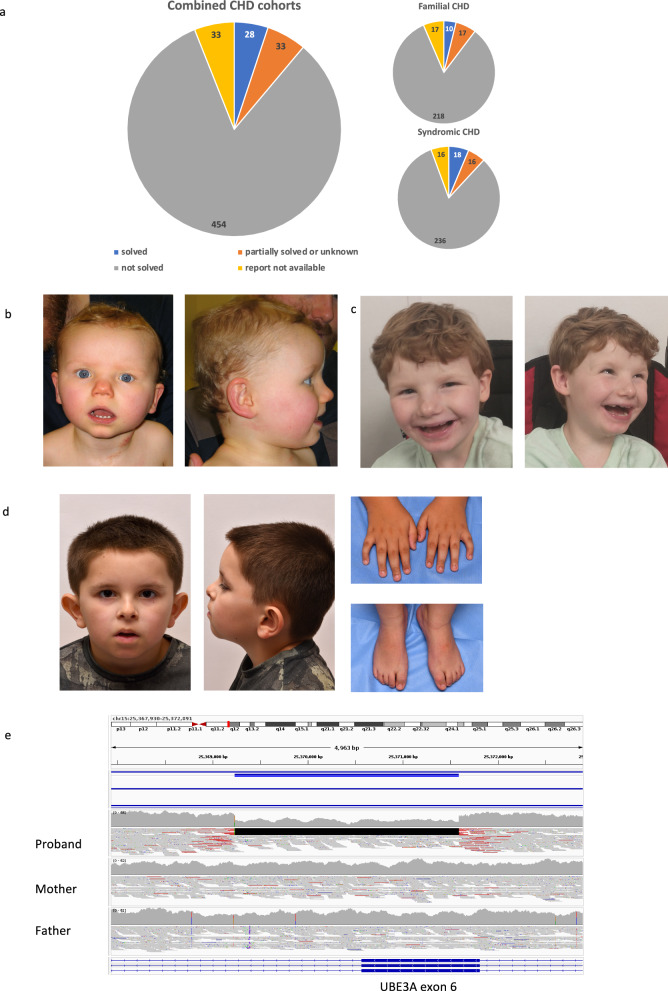
A summary of the outcome for participants of the CHD cohorts of 100kGP and images of participants with pathogenic or likely pathogenic variants identified by our GDP. **a** Pie-charts quantifying the number of participants within our syndromic and familial CHD cohorts, as well as the combined cohorts, classified by GEL as “solved” (blue), “partially solved or unknown” (orange), “unsolved” (grey) or “report not available” (yellow). **b** Facial photographs of participant #9 at age 1 year 7 months, showing down-turned mouth and widely spaced teeth. **c** Facial photographs of participant #9 at age 8 years, showing happy demeanour, wide mouth, wide nasal bridge and widely-spaced teeth. **d** Photographs of participant #5 at age 7 years showing a broad forehead, brachycephaly, deep set eyes and broad nasal bridge. Hand and foot images show brachydactyly, 5th finger clinodactyly and tapering fingers. **e** IGV screenshot showing de novo deletion identified in Participant #9. The proband’s aligned WGS reads from .VCF file are compared to those of both parents. The deletion is shown to affect part of exon 6 of *UBE3A*.

From the 100kGP, we identified a cohort of 286 syndromic CHD (sCHD) probands and 262 familial CHD (fCHD) probands with GRCh37 or GRCh38-aligned genomes available. Of these participants (including those recruited to the sCHD and fCHD categories), 328 were submitted as trios with both parent samples available. Within the cohort, many different cardiac phenotypes were represented, including septal defects (52.4%), valvular defects (40.5%), tetralogy of Fallot (11.2%), and transposition of the great arteries (7.7%). A comprehensive overview of the cardiac phenotypes seen in this cohort are provided in Supplementary Table 1. Around half of the identified participants had “syndromic” disease (CHD plus neurodevelopmental delay or extra cardiac malformation or both, and no recognized syndrome), and 77.68% had one or more non-cardiac feature recorded in the HPO terms. Overall, 6.29% of the sCHD cohort and 3.89% of fCHD cohort were recorded as “solved” (Fig. 2a). In total, *n* = 39 likely pathogenic or pathogenic variants and *n* = 56 VUS were identified.

### The gene discovery pipeline provided diagnostic uplift in CHD cohorts

Nine heterozygous de novo variants (DNVs) in nine different known genes were identified as a potential or likely cause of disease by our GDP (Table 1), comprising eight SNVs and one SV. The ACMG classification of variants awarded by the research team is given in Table 1. For a full description of ACMG classifications, see Supplementary Table 2. Seven variants were classified as “pathogenic” or “likely pathogenic” (and were therefore included in our calculation of uplift) and two variants were VUS. The most common factors for a missed diagnosis were: 1) the morbid gene was not included on the applied gene panels (i.e. when panels were applied according to phenotype, they did not include the disease gene); or 2) the disease gene is newly-identified and is not represented on the applied gene panel (i.e. this is a new disease-gene subsequently included on the applied panel).

**Table 1:** Summary of 9 cases of diagnostic uplift where filtered DNVs affected a morbid-gene, consistent with participant phenotype.

Research Patient no.	Category	Phenotype (raised HPO term)	Gene	Variant; HGVSc (GRCh38)	Variant HGVSp	Variant type	SIFT	Polyphen	CADD	GnomAD	ACMG Classification	MIM	Tier	Exomiser position	Reason not reported
1	Syndromic CHD	Radioulnar synostosis, abnormality of the pulmonary vasculature, mitral valve prolapse, SNHL, VSD, tall stature	*MECOM*	3:169100903 G > A	NP_004982.2: T944I	Missense variant	D(0)	PD (0.999)	31	absent	LP	616738	–	1	Added to applied panel after analysis
2	Syndromic CHD	abn ventricular septum morphology, displacement of the urethral meatus, abn location of the ears, abn aortic morphology, congenital malformation of the right heart.	*ZIC3*	X:137567718 G > A	NP_003404.1: E343K	Missense variant	D(0)	B (0.358)	25.4	absent	LP	306955	2	1	Had been tiered by GEL and was reported subsequently
3	Familial CHD	CHD, developmental delay, learning disability, feeding difficulty	*SETD5*	3:9447906 C > G	NP_001073986.1: S668*	Stop gained	–	–	35	absent	P	615761	3	1	Not on applied panels
4	Syndromic CHD	Aortic root abnormality, abn of the musculoskeletal system, abn palate morphology, abn ventricular septum morphology	*EHMT1*	9:137784146 G > A	NP_001138999.1: W808*	Stop gained (final exon)	–	–	2.22	absent	VUS	610253	3	2	Not on applied panels
5	Syndromic CHD	CHD, Failure to Thrive, neurological features	*CNOT2*	12:70319362 AAAGT > A	NP_055330.1: S80Cfs*57	Deletion	–	–	–	absent	P	618608	3	5	Added to applied panel after analysis
6	Syndromic CHD	congenital malformation of the left heart, headache	*MYRF*	11:61771657 C > T	NP_001120864.1: Q300*	Stop gained	–	–	36	absent	P	618280	3	2	Not on applied panels
7	Syndromic CHD	CHD, Failure to Thrive, neurological features	*ARID1A*	1:26772816 A > T	NP_006006.3: S1182C	Missense variant	D (0.02)	PD (0.993)	28.1	absent	VUS	614607	3	6	Not on applied panels
8	Syndromic CHD	CHD	*CHD4*	12:6583240 G > A	NP_001264.2: R1340C	Missense variant	D (0.02)	PD [1]	32	absent	LP	617159	3	2	Not on applied panels
9	Familial CHD	microcephaly, ASD, developmental delay, abn mitral valve. HLHS	*UBE3A*	15:25369233-25371590del	NP_570854.01: Cys195Serfs*21	Deletion	–	–	–	–	P	105830	–	–	CNV, not identified

*Abn* abnormal, *ASD* Autistic Spectrum Disorder, *B* Benign, *CHD* Congenital Heart Disease, *CNV* Copy Number Variant, *D* Deleterious, *GEL* Genomics England, *HLHS* Hypoplastic Left Heart Syndrome, *LP* Likely Pathogenic, *P* Pathogenic, *PD* Probably Damaging, *SNHL* Sensorineural Hearing Loss, *VSD* Ventricular Septal Defect, *VUS* Variant of Uncertain Significance.

Participant #1 was a 19-year-old man with Eisenmenger syndrome, a large perimembranous VSD and pulmonary hypertension. He had a dysplastic aortic valve and tortuous pulmonary veins. He had bilateral sensorineural hearing loss and radio-ulnar synostosis (RUS) (Supplementary material). MRI of the brain had shown skull haemangiomas. He had detrusor instability, but no urogenital abnormality. He has no history of developmental delay and is of normal intellect. At the age of 11-years he suffered an acute inferior myocardial infarction due to an embolus. He went on to have a heart and lung transplant aged 15 years. Post-transplant he developed lymphoproliferative disease. On examination he is not facially dysmorphic, but has bilateral 5th finger clinodactyly, short thumbs and right sided radio-ulnar synostosis.

The participant was entered into the sCHD cohort of 100kGP and no variants were reported from the initial pipelines. Our GDP identified a heterozygous de novo missense variant in *MECOM;* (HGNC 3498), GRCh38; chr 3:169100903 G > A, *MECOM*(ENST00000651503.2):c.2831 C > T, (p.Thr944Ile). In silico tools supported the pathogenicity of this variant. Variants in *MECOM* cause Radioulnar Synostosis with Amegakaryocytic Thrombocytopenia-2 (RUSAT2; OMIM 616738). This missense variant occurs within the 8th zinc finger (ZF) motif region of the protein, as described for other missense variants causing RUSAT [23]. This participant did not demonstrate any haematological manifestations of the condition, which is consistent with reports of an absence of haematological findings in some patients with RUSAT with missense variants in the 8th and 9th ZF motifs [24]. CHD (e.g. septal defects, aortic coarctation and tetralogy of Fallot) is a known additional feature of this condition [25]. The local diagnostic laboratory issued a report of likely pathogenic classification.

Participant #5 had known developmental delay, conductive hearing loss, aortic stenosis and bicuspid aortic valve with aortic regurgitation. On examination he had a “stocky” build, broad forehead, brachycephaly, deep set eyes, broad nasal bridge and two extra accessory nipples on both sides of the chest (see Fig. 2d and Supplementary material). This participant was entered into the sCHD cohort of 100 kGP and a negative result was issued. Our study identified a heterozygous de novo 4 bp deletion in *CNOT2; (*HGNC 7878) 12:70319362: AAAGT > A, (ENST00000229195.8):c.238_238+2del; p.Ser80Cysfs*57, predicted to result in a frameshift, consistent with the loss-of-function mechanism of disease. The participant had facial and skeletal features consistent with *CNOT2*-related disorder (IDNADFS, OMIM 618608), and supernumerary nipples have been previously reported in the condition [26]. The diagnostic laboratory classified this variant as likely pathogenic. CHD (for example, ventricular septal defects, pulmonary stenosis and dysplastic valves) is known to occur in patients with CNOT2-related disorder and so this finding was consistent with his cardiac presentation [26].

One participant received a diagnosis through identification of an SV using the SVRare script. Participant #9 (Fig. 2b, c and Supplementary material) was an 8-year-old girl with HLHS, severe developmental delay, speech delay, intellectual impairment, ataxic gait and feeding difficulties. Examination revealed she had an ataxic gait, increased lower limb tone with brisk deep tendon reflexes, central hypotonia and increased drooling. She had microcephaly (head circumference <0.4th centile, *z* score -3.9, weight 16th centile, and height 6th centile). She had flattened occiput, intermittent squint, and wide mouth (Fig. 2b, c). Angelman syndrome was clinically suspected prior to genomic testing.

CGH microarray and methylation-specific PCR for Angelman syndrome were normal. She was enrolled into the 100kGP fCHD category, and initial analysis was negative. This study identified a heterozygous 2.3 kb de novo deletion in *UBE3A;* (HGNC 12496) (NM_130839.5): c.584_1608+1333del; p.Cys195Serfs*21 (Fig. 2e). This had not previously been identified on the original BlueGnome 8x60k v2.0 ISCA platform array due to incomplete probe coverage. The deletion was subsequently confirmed with targeted array analysis using the Illumina GSAv3 microarray. The small size of this deletion could only be detected after removal of the usual 1 kb filter. This variant was predicted to result in a premature termination codon and loss of protein function. Deletions of *UBE3A* have been demonstrated to cause Angelman syndrome, in the absence of methylation abnormalities [27]. However, Angelman syndrome is not typically associated with CHD and this finding may indicate an expansion of the known phenotype. The variant has been confirmed and reported as likely pathogenic by the local diagnostic laboratory.

### Candidate gene were prioritised according to gene constraint and expression scores

Candidate genes identified by the GDP (*n* = 79) were added to a dataset and annotated for prioritisation. The top candidate genes from our data set, when ordered for percentile rank using expression in developing mouse heart at E14.5, are shown in Table 2. The high percentile rank for expression of these genes in developing mouse heart indicates a key role in cardiac development. The full list of prioritised candidate genes (*n* = 79), alongside annotation values, is available in Supplementary Table 3.

**Table 2:** Candidate disease genes from the gene prioritisation dataset; the most highly expressed genes in mouse fetal heart tissue at E14.5.

Patient research number	Gene	HUGO ID	Variant Consequence	Gnomad AF	SIFT	Polyphen	CADD score	LOEUF score	GTEX expression (median TPM)	Rank mouse heart expression E14.5
10	CCT2	HGNC:1615	Missense	Absent	Deleterious (0.01)	Probably damaging (0.984)	29.2	0.23	38.83	98.6
11	*APLP2*	HGNC:598	Missense	Absent	Deleterious(0)	Probably damaging (0.999)	24.4	0.39	99.92	98.5
12	*PYGB*	HGNC:9723	Missense	Absent	Tolerated (0.11)	Benign(0)	22.5	0.89	100.2	97.9
13	*KIAA0100*	HGNC:28960	Missense	Absent	Tolerated (1)	Benign (0.056)	17.1	0.42	17.39	95.4
14	*AKAP13*	HGNC:371	Missense	Absent	Deleterious (0.03)	Probably damaging (0.998)	29.2	0.29	22.68	94.4
15	*ZC3H4*	HGNC:17808	Missense	Absent	Tolerated (1)	Probably damaging (0.997)	21.8	0.054	5.264	89.8
16	*SIPA1L1*	HGNC:20284	Missense	Absent	Tolerated(0.79)	Benign (0.074)	17.75	0.17	1.071	88.7
17	*GOLGA6A*	HGNC:32206	Frameshift	Absent	–	–	–	1.18	0	88.4
18	*UBR2*	HGNC:21289	Missense	Absent	Tolerated (0.36)	Benign (0.009)	18.66	0.21	7.637	87.3
19	*NUP98*	HGNC:8068	Missense	Absent	Tolerated (0.27)	Possibly damaging (0.544)	23.4	0.12	9.499	87.2
20	*HMCN1*	HGNC:19194	Missense	Absent	Tolerated (0.12)	Benign (0.003)	15.55	0.48	0.923	85.9

The mouse expression data was derived from RNA sequencing of tissue from E14.5 mouse embryos (for published data and methodology see [10, 19]). Datasets were from the following tissues: left and right atria, left ventricle (with interventricular septum, aortic and mitral valves), and right ventricle (with pulmonary and tricuspid valves). The average of rpm (reads assigned per million mapped reads) of each gene from each chamber was used as the measure of overall heart expression. The table lists: Patient research number, Gene name, HUGO ID, variant features (including in silico tool prediction outcomes), LOEUF score, human heart GTEX expression and ranked mouse heart expression at E14.5.

*AF* allele frequency.

### Candidate variants were assessed in patients with CHD from an additional cohort, but no additional DNVs were identified

In total 12 candidate variants, in genes *HMCN1, CBLB, UBR2, NR4A1, AHNAK2, TFAP2C, KIAA0100* and *AKNA* were identified in probands with CHD in the additional cohort. On segregation, all variants were found to be inherited from an unaffected parent (supplementary Fig. 1). However, these findings do not exclude pathogenicity of the variants due to the possibility of incomplete penetrance.

### Candidate CHD genes include those implicated in cardiac development and novel functions

Our prioritised candidate gene list contained genes known to be involved in cardiac development e.g., *AKAP13* (HGNC 371) and *BCAR1* (HGNC 971) (Supplementary Table 3 and 4). Supplementary Table 3 shows the full list of prioritised candidate CHD genes. Those genes considered by the study team of highest importance for follow-up, based on their known function or expression pattern, are listed in Supplementary Table 4.

Cardiomyocytes of *Akap13*-null mice have deficient sarcomere formation and thin-walled developing hearts [28]. *BCAR1* (HGNC 971) is essential for ventricular development and neural crest cell remodelling of the outflow tract [29]. Both genes are highly expressed in both human heart tissue (GTEx median expression 22.68 TPM and 15.7 TPM, respectively) and mouse fetal heart tissue (percentile ranked expression levels at E14.5 of 94.4 and 82.63, respectively).

Rare variants in *HMCN1* (HGNC 19194) were previously reported in association with pulmonary atresia (PA) [30]. However, familial studies were not performed in this report, so de novo status is unknown. In our study we identified a de novo *HMCN1* variant in a participant in the CHD dataset, providing additional evidence for the association with CHD, and showing the utility of our methodology. *HMCN1 (FBLN6)* encodes hemicentin-1, an essential component of extracellular matrix, known to be important in the formation of cell-cell contacts, as well as cardiac fibroblast migration, in response to the TGF-beta signalling pathway [31]. HMCN1 deficiency is associated with defects in cell migration in nematodes and is early embryonic lethal in mice [31], and so is a highly plausible CHD candidate gene.

*SETDB1* (HGNC 10761) encodes a histone methyltransferase. DNVs in genes associated with histone methylation are commonly associated with CHD [19], and *SETDB1* could therefore represent a novel disease gene.

A DNV in *TFAP2C* (HGNC 11744), encoding transcription factor AP-2 gamma, was identified in one participant (Supplementary Table 3). TFAP2C expression is controlled by CITED2 (a transcription co-activator). CITED2 is involved in left-right patterning and variants in this gene cause CHD, conotruncal defects and laterality defects [32], making *TFAP2*C a plausible functional candidate gene for CHDs. However, expression levels were not high (median expression in human left ventricle; 0.093 TPM, percentile ranked expression level at mouse E14.5; 15.04).

## Discussion

A total of 28 participants recruited to the identified CHD cohort were reported by GEL as “solved” (18 from the sCHD cohort and 10 in the fCHD cohort), with a further 33 cases categorised as “partially solved” or “unknown”. There was no recurrence of causative genes, demonstrating the genetic heterogeneity of CHD.

### Diagnostic uplift

Within the CHD participants, 95 pathogenic (P) variants, likely pathogenic (LP) variants or variants of uncertain significance (VUS) were reported from the GEL pipelines. In this study, 9 additional DNVs were identified, all potentially clinically important. Identification of the causative variant for patients with rare disease allows clinicians to provide prognostic and monitoring information for families, as well as allowing for counselling on recurrence risk and future prenatal options.

The approach used here demonstrates the utility of a “reverse phenotyping” approach, meaning that potentially pathogenic variants were identified first, with no clinical bias. This reverse phenotyping strategy has been successful in other heterogenic conditions such as ciliopathies [11]. This study confirms the importance of DNVs in the pathogenesis of CHD, particularly in the context of syndromic disease. However, our identification rate of DNVs is lower than in previous publications looking at “syndromic” CHD (for example [33, 10]), where approximately 20% of patients had a damaging DNV identified. This may be due to the different recruitment populations studied, and in this study we have excluded participants who received a diagnosis through the 100kGP core pipelines. This work supports an agnostic DNV analysis approach to GS data analysis in unsolved cases, for which DNVs are assessed outside of gene panels to increase the diagnostic yield.

Interestingly, not all of the identified diagnoses were conditions typically related to CHD (for example Angelman syndrome), which suggests either this study expands the known phenotype, or that the CHD was co-incidental to the other challenges of the participant. One could argue that in some instances, recruitment to the CHD cohort of the 100kGP was not the most appropriate recruitment category for patients with syndromic disease or intellectual disability, as there were five instances identified here for which the causative gene was not on the applied panels. In some cases, the phenotypic data submitted to the 100 kGP was sparse, so making genotype-phenotype correlation challenging [34].

The majority, 89% (*n* = 8/9), of identified variants associated with diagnostic uplift in this study were within the top 10 hits in Exomiser (Table 1). Exomiser is a Java program that identifies potential disease-causing variants from GS data using HPO terms [35]. Exomiser appears a useful adjunct to diagnostic pipelines, however, because Exomiser utilises phenotypic data, this is also dependent upon accurate HPO term entry. Furthermore, the importance of reanalysis of data over time is clear, as new disease genes are added to panels. Our results would support an automated process to re-analyse GS data at regular intervals, and there is some evidence that such approaches improve diagnostic yield [36].

The 100 kGP pipelines did include an SV reporting feature, which involved tiering of calls from the Canvas copy number caller, but this was only introduced towards the end of the project [14]. CNV calls were also made and recorded by the Manta copy number caller, but were not considered in the tiering pipeline [13]. The use of the SVRare scripts in this study demonstrates the need for thorough CNV analysis in GS data assessment. The methodology of the SVRare scripts (exclusion if the call occurs in >10 100kGP participants) appears to be an effective cut-off [12].

### Candidate CHD genes

Our methodology has also allowed the creation of a dataset of candidate CHD genes. These can be prioritised according to gene expression scores and gene constraint.

Screening of a separate cohort of patients with mixed types of CHD identified rare variants in the candidate genes, but a second DNV was not identified. The identification of rare variants in the gene *HMCN1* in additional patients with pulmonary atresia from the medical literature, supports the study findings. Due to the known function of this gene and its links to the cardiac developmental process, including cardiac fibroblast migration, our findings provide further evidence for the role of *HMCN1* in CHD pathogenesis, supporting the utility of our methodology. However, the putative gene-disease associations suggested by the data are not yet proven and further studies are required to confirm the pathogenicity of these variants. The study team plan to further investigate the potential importance of these candidate genes, using tools such as Decipher [37] and Gene Matcher [38], for the benefit of the study participants and other CHD patients worldwide.

## Conclusions

In this study, our de novo Gene Discovery Pipeline has provided diagnostic uplift of nearly one third over results reported by 100kGP, providing clinical results that were extremely important to patients and families. The research study has also created a dataset of genes that are functional candidate CHD disease genes, but further confirmation is required in additional cohorts of patients affected with CHD. Our methodology has likely utility for functional candidate gene identification in other phenotypic categories within 100kGP, as well as other large scale genome sequencing projects, highlighting the opportunities and research benefits associated with large GS research studies.

## Supplementary information

Supplementary Material File

Supplementary Table 1

Supplementary Table 2

Supplementary Table 3

Supplementary Table 4

Supplementary Figure 1

Supplementary Material

## Data Availability

The data supporting the conclusions of this article are included within the article and its additional files. Further data of this study may be available from the corresponding author upon request. Research on the de-identified patient data used in this publication can be carried out in the Genomics England Research Environment subject to a collaborative agreement that adheres to patient led governance. All interested readers will be able to access the data in the same manner that the authors accessed the data. For more information about accessing the data, interested readers may contact research-network@genomicsengland.co.uk or access the relevant information on the Genomics England website: https://www.genomicsengland.co.uk/research.

## Code availability

The code used in this study is available online at GitHub. (https://gist.github.com/matthewroche/bc3aac244f8007bbb93afc4f91dd0675). The data used in this study are available to registered users within a protected research environment and information about this can be found at https://www.genomicsengland.co.uk/about-gecip/for-gecip-members/data-and-data-access/.
