# Supplementary material for: Molecular diagnoses and candidate gene identification in the congenital heart disease cohorts of the 100,000 genomes project
Source: Eur J Hum Genet. 2024 Nov 26;33(6):793–802. doi: 10.1038/s41431-024-01744-2 (PMC12185689; doi:10.1038/s41431-024-01744-2)
Supplement: Supplementary file 1 — Supplementary Material File [file 41431_2024_1744_MOESM1_ESM.docx]

**Supplementary Material**

Detailed case report associated with diagnostic uplift: *MECOM*

Participant #1 is a 19-year old man who was diagnosed with Eisenmenger syndrome aged 2 years 6 months of age, secondary to a large perimembranous VSD and pulmonary hypertension. He was also found to have a dysplastic AV valve and an unusual tortuous course of the pulmonary veins. He was born at term by NVD with no known prenatal issues. His birth weight was 7lb 8oz. He was diagnosed with moderate bilateral sensorineural hearing loss aged 3 years and was myopic. MRI brain had shown skull haemangiomas. He had detrusor instability but no urogenital abnormality. He has no history of developmental delay and is of normal intellect. At the age of 11-years he suffered an acute inferior myocardial infarction due to an embolus. He went on to have a heart and lung transplant aged 15 years. Post-transplant he developed lymphoproliferative disease. On examination he is not facially dysmorphic, but has bilateral 5th finger clinodactyly, short thumbs and right sided radio-ulnar synostosis. At the age of 14 years his head circumference was on the 85th centile and height on the 93rd.

Previous testing had included a normal microarray, and normal *SALL1/4* and *TBX5* testing. The proband was entered into the syndromic CHD cohort of the 100kGP as a trio with his parents, with the applied gene panels “familial non syndromic congenital heart disease”, “hearing loss”, “limb disorders” and “thoracic aortic aneurysm or dissection”. No variants were reported from the initial pipelines. This study identified a *de novo* missense variant in *MECOM*, GRCh38; chr 3:169100903 G>A, *MECOM*(ENST00000651503.2):c.2831C>T, (p.Thr944Ile), which was absent from GnomAD and had a CADD score of 31. SIFT and Polyphen *in silico* prediction tools predicted a damaging effect of the variant on the protein. This variant was reported to the recruiting clinician and was felt to be consistent with his clinical features. The local diagnostic laboratory confirmed the finding by Sanger sequencing and issued a report of Likely Pathogenic classification.

Detailed case report associated with diagnostic uplift: *CNOT2*

Participant #5 is the 2^nd^ child of a non-consanguineous couple. Both parents reportedly have mild dyslexia. His elder sister has mild learning difficulties but no developmental concerns or medical problems. In the pregnancy, his mother had a small bleed at 13-14 weeks. This participant was born by planned LSCS at 39 weeks for breech position. No resuscitation was required. The participant was observed in SCBU for feeding difficulties and hypoglycaemias, and later developed gastroesophageal reflux.

This proband had developmental delay: he first sat up with support at 8 months, independently at 12 months, and walked at 18 months. He was noted to be very hypermobile and “clumsy”. He required physiotherapy input, but there were no significant concerns about his fine motor skills. He had significant delay in expressive verbal language with no speech at 2-and-a-half-years old. He compensated by using a wide range of gesture, some Makaton and some sign language, pointing, facial expressions and eye contact. He had been under the care of a speech and language therapist. He had good and engaging social skills. He was diagnosed with congenital conductive hearing loss and uses hearing aids.

This proband was noted to have a heart murmur at age 3 when assessed for his speech and motor delay. He was diagnosed with aortic stenosis and bicuspid valve with regurgitation. He had on-going walking difficulties and wears shoes with an insole. He was recently noted to have brisk deep tendon reflexes and is due an MRI scan of the brain.

On examination his height was 9^th^ percentile, weight 75^th^ percentile, and head circumference 25^th^ percentile. He had a “stocky” build, broad forehead, brachycephaly, deep set eyes, broad nasal bridge and two extra accessory nipples (on both sides) (see Fig 1d). MRI brain was normal, but a recent ophthalmology review raised the possibility of optic nerve hypoplasia and so he is having further investigations for this.

The proband and both parents were recruited to the 100kGP, but the initial project gave a negative result. *De novo* analysis in this study identified a 4 bp deletion in *CNOT2* 12:70319362: AAAGT>A, (ENST00000229195.8):c.238_238+2del; p.Ser80Cysfs*57, predicted to result in a frame-shift that is consistent with the loss-of-function mechanism of disease. The diagnostic laboratory subsequently classified this variant as Likely Pathogenic (LP) and this was felt to be consistent with his phenotype, confirming a diagnosis of *CNOT2*-related disorder.

Detailed case report associated with diagnostic uplift: *UBE3A*

Participant #9 was an 8-year old girl with hypoplastic left heart syndrome, severe developmental delay, speech delay, intellectual impairment, microcephaly, ataxic gait, squint, infantile feeding problems and a behavioural phenotype including poor sleep, sensory behaviours and excitable behaviour. She was born at term in good condition with a weight of 3.3kg (40th centile) to healthy non-consanguineous parents. Her antenatal scans were normal. The hypoplastic left heart syndrome was diagnosed shortly after birth and she underwent a 3-stage surgical repair which concluded with a palliative Fontan procedure at the age of 6. She did not have seizures and she has not had an MRI brain scan or EEG.

Examination revealed she had an ataxic gait with an uplifted, flexed arm position, increased lower limb tone with brisk deep tendon reflexes, central hypotonia and increased drooling. She had microcephaly (head circumference <0.4th centile, z score -3.9, weight 16th centile, height 6th centile), flattened occiput, intermittent squint, wide mouth, wide nasal bridge and widely spaced teeth (Fig 2a and b). She had auburn hair and blue eyes. She exhibited frequent spontaneous laughter and smiling, hand flapping and good eye contact.

CGH microarray and methylation specific PCR for Angelman syndrome at age 1 year 7 months revealed no evidence of clinically significant copy number variant, deletion, disomy or imprinting defect. She was enrolled into the 100,000 Genomes Project (100kGP). Testing for genes on the gene panels for primary ciliary disorders, familial non-syndromic congenital heart disease and intellectual disability did not identify a candidate variant. The 100kGP initially reported no pathogenic findings.

*De novo* variant (DNV) in study identified a heterozygous pathogenic 2.3kb *de novo* deletion in UBE3A (NM_130839.5): c.584_1608+1333del; p.(Cys195Serfs*21) (Fig 2c). The variant was predicted to result in a premature termination codon and loss of protein function. It has not been reported in the gnomAD population database. This variant was reported as pathogenic by the local diagnostic laboratory and allowed the family to reach a conclusive diagnosis for their child, as well as allowing for the possibility of future reproductive options.
