# Supplementary material for: Molecular diagnoses and candidate gene identification in the congenital heart disease cohorts of the 100,000 genomes project
Source: Eur J Hum Genet. 2024 Nov 26;33(6):793–802. doi: 10.1038/s41431-024-01744-2 (PMC12185689; doi:10.1038/s41431-024-01744-2)

HMCN1 Ile785Lys

proband	mother	father
T003156	T003182	T003183
Het	Het	WT(T)

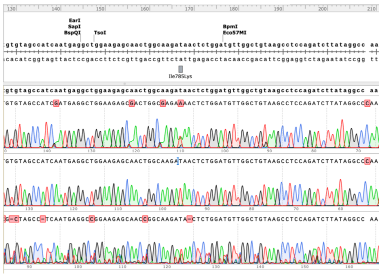

HMCN1 Glu2915Lys

proband	mother	father
T002336	T002335	T002334
Het	WT (G)	Het

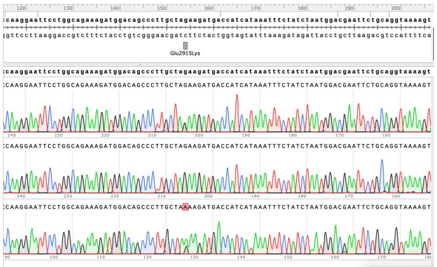

HMCN1 Ala4136Thr

proband	mother	father
T002511	T002512	T002513
Het	Het	WT (G)

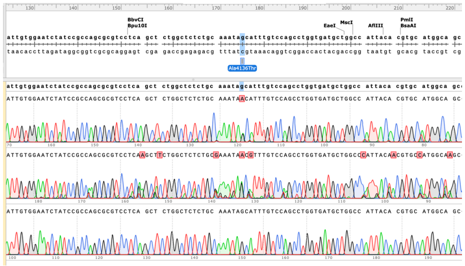

CBLB Ser614Pro

proband	mother	father
T001061	T001055	T001074
Het	Het	WT (A)

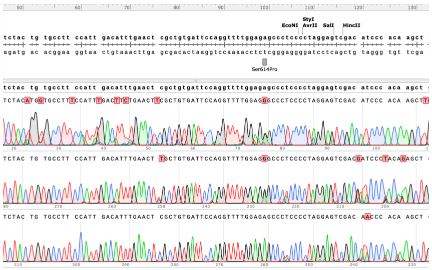

CBLC Tyr106Ser

proband	mother	father
T001064	T001065	T001240
Het	Het	WT (T)

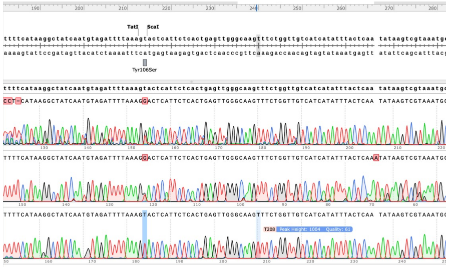

UBR2 Glu1565Ter

proband	mother	father
T001557	T001549	T001547
Het	WT (G)	Het

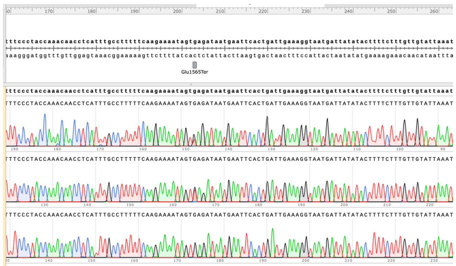

NR4A1 Thr55Asn

proband	mother	father
T003113	T002936	T002937
Het	WT (C)	Het

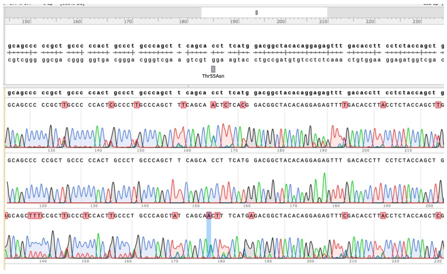

NR4A1 Ala452Thr

proband	mother	father
T002953	T002641	T002642
Het	WT (G)	Het

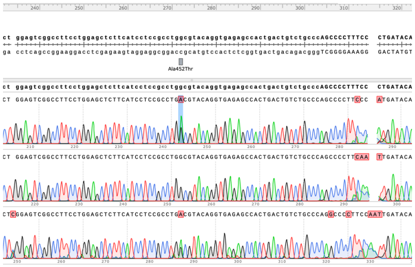

AHNAK2 Asp4125Gly

proband	mother	father
T002617	T002618	T002619
Het (TGC)	Het (TGC)	WT

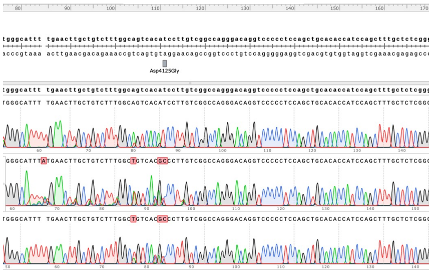

AKNA Glu897GlyfsTer58

proband	mother	father
T003220	T003232	T003233
Het	Het	WT (T)

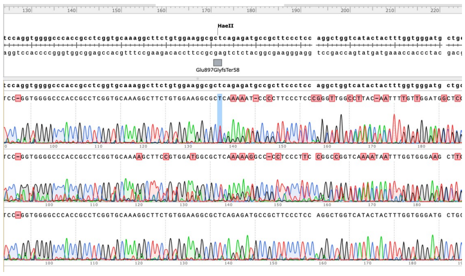

TFAP2C Pro44Ser

proband	mother	father
T002442	T002443	T002444
Het (T)	WT	Het (T)

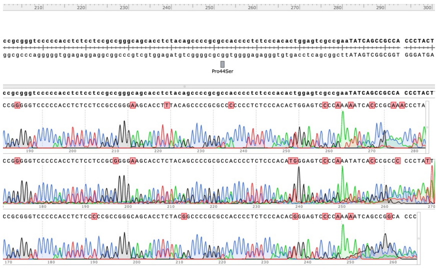

KIAA0100 Gln517Ter

proband	mother	father
T003802	T003803	T003804
Het (A)	Het (A)	Het (A)

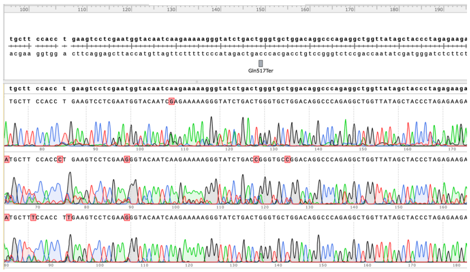

Supplement: Supplementary file 6 — Supplementary Figure 1 [file 41431_2024_1744_MOESM6_ESM.pdf]
