# Supplementary material for: Molecular diagnoses and candidate gene identification in the congenital heart disease cohorts of the 100,000 genomes project
Source: Eur J Hum Genet. 2024 Nov 26;33(6):793–802. doi: 10.1038/s41431-024-01744-2 (PMC12185689; doi:10.1038/s41431-024-01744-2)
Supplement: Supplementary file 7 — Supplementary Material [file 41431_2024_1744_MOESM7_ESM.docx]

**Supplementary Material**

**Supplementary Material:** **Detailed case reports of three participants from the 100kGP where a DNV was identified as the genetic cause for their condition in this study.**

**Supplementary Table 1: A detailed phenotypic analysis of the CHD cohorts studied.** The table columns indicate the phenotypic feature identified in the cohort, the number of participants with a corresponding HPO term, and the percentage of the total cohort studied with that feature.

**Supplementary Table 2: ACMG classification of variants identified in the participants listed in Table 1.** The dataset provides a detailed ACMG analysis of the identified *de novo* variants.

**Supplementary Table 3: Non-morbid genes in which DNVs were identified after filtering of the CHD “unsolved” cohort.** Columns list: variant type, gene name, HUGO gene number, CADD Phred score of variant, presence in GnomAD database, SIFT score of variant, PolyPhen score of variant, LOEUF score of gene, GTEX expression score (heart; left ventricle, TPM) from deceased human individuals and E14.5 mouse heart rank percentile expression figure (Homsy et al. 2015). Those genes with a known or proposed cardiac function are indicated by a cross in the final column.

**Supplementary Table 4: The novel genes considered to be high priority for follow-up by the research team.** The novel gene list (Supplementary Table 3) has been filtered to demonstrate those genes considered by the research team to be highest priority for follow-up. These include genes with a known or predicted role in cardiac development and/ or a very high expression level in mouse embryonic heart tissue (>90 percentile rank). Columns are; variant type, gene name, HUGO gene number, CADD Phred score of variant, presence in GnomAD database, SIFT score of variant, PolyPhen score of variant, LOEUF score of gene, GTEX expression score (Heart; left ventricle, TPM) from deceased human individuals and E14.5 mouse heart rank percentile expression figure (Homsy et al. 2015). Those genes with a known or proposed cardiac function are indicated by a cross in the final column.

**Supplementary Figure 1: Sanger sequencing electopherograms showing segregation of rare variants in candidate genes in trios (proband and their unaffected parents) from the additional CHD patient cohort.** All assessed rare variants were inherited from at least one unaffected parent.
